# The severity of the second wave of SARS CoV-2 in maintenance dialysis patients of a single center in Brazil

**DOI:** 10.1080/0886022X.2022.2069580

**Published:** 2022-05-04

**Authors:** Fernanda Salomão Gorayeb-Polacchini, Heloisa Cristina Caldas, Mario Abbud-Filho

**Affiliations:** a Nephrology Division, Medical School FAMERP & Dialysis Unit, Hospital de Base/FUNFARME, Sao Jose do Rio Preto, Brazil; b Laboratory of Immunology and Experimental Transplantation (LITEX), Medical School FAMERP, Sao Jose do Rio Preto, Brazil

Dear Editor,

Maintenance dialysis patients have higher severity and mortality from SARS-CoV-2 infection compared to the general population [1,2]. In addition, they have a higher risk of infection due to the need to travel to dialysis units and frequent contact with other patients and health professionals [1].

Brazil is among the three countries with the highest number of confirmed cases and deaths due to COVID-19 and globally it is ranked in third considering the number of chronic dialysis patients, with approximately 92% of in-center hemodialysis [2,3].

The first COVID-19 case in Brazil was detected on 26 February 2020, in the city of São Paulo. Until January 2022, more than 22 million confirmed cases with over 610,000 deaths due COVID-19 were reported in Brazil [3].

Since the end of 2020, there has been a great concern about the variants of SARS-CoV-2; the B.1.1.7 variant identified in the United Kingdom, the B.1.351 variant discovered in South Africa, and the P.1 variant classified as a ‘variant of concern’ *Gamma* originating from the Brazilian state of Amazonas. The three variants were associated with an increase in transmissibility and worsening of the epidemiological situation in the regions where they expanded [4].

The variant *Gamma* was first identified in travelers from Japan returning from the state of Amazonas (Brazil), causing in January 2021 an early second wave in Amazonas state compared to the rest of Brazil. A study reporting the second wave of COVID-19 in Amazonas shows that there is an increase in the number of hospitalizations and mechanical ventilation need, when compared to the first wave [4].

**Figure 1.**
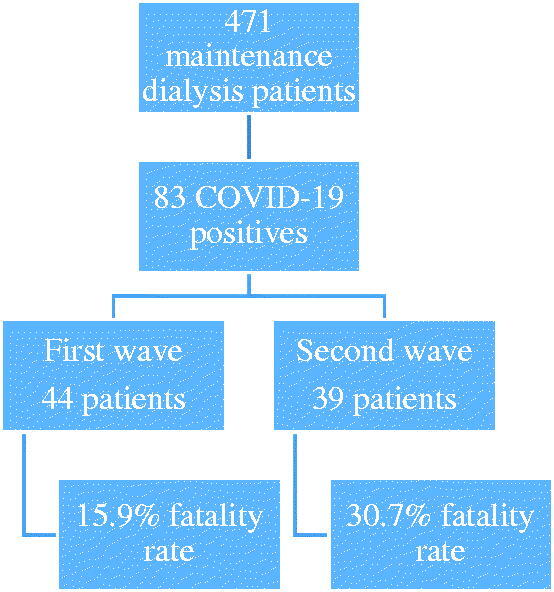
Study design.

We evaluated 441 and 360 maintenance dialysis patients in the first and second waves respectively, occurring between May 2020 and May 2021, those presenting symptoms or history of close contact with COVID-19 were tested with nasopharyngeal swab RT-PCR. COVID-19 positive patients were included in the study (Figure 1). The study protocol was approved by the Medical School (FAMERP) Ethics Committee (# 4212395). We sought to compare the outcomes of the COVID-19 disease of the first (from 1 May 2020 to 31 January 2021), and the second wave (from 1 February 2021 to 1 May 2021) in a single center of dialysis in Brazil. Our dialysis facility is a regional reference center for the treatment of end stage renal disease (ESRD) gathering around 2 million inhabitants. We evaluated the demographic characteristics and the incidence, severity of the disease, mortality rates, and case fatality rates during the first and second waves.

We classified it as a severe disease, one of the following: patients with computed chest tomography presenting more than 50% of ground glass lesions, intensive care unit hospitalization need, respiratory failure requiring mechanical ventilation (MV), or death. The incidence, mortality, and case fatality rates were calculated as follows: incidence = number of COVID-19 cases/number of exposed people (dialysis patients) per 10,000. Mortality = number of deaths due to COVID-19/number of exposed people (dialysis patients) per 10,000. Fatality = (number of confirmed deaths due to COVID19/number of confirmed COVID-19 cases) *100. Quantitative variables (such as age and duration of treatment) are presented as mean ± SD, while categorical variables (such as sex and treatment modality) are presented as frequencies (percentage). The Shapiro–Wilk test was used to verify if clinical data were normally distributed, and values were compared using parametric (Student’s *t* test) or equivalent nonparametric tests (Mann–Whitney *U* test). Associations among the categorical variables were expressed by the Chi-square test or by the Fisher exact test. Data analyses were performed using the StatsDirect 3.0 software and *p* < 0.05 values were considered indicative of statistical significance.

The maintenance dialysis patients infected with COVID-19 were predominantly male (59%), with a mean age of 60 ± 15 years. The mainly primary causes of ESRD were diabetes mellitus (39.7%), followed by arterial hypertension (31.1%) (Table 1). Fever (65%), cough (62.6%), and dyspnea (53%) were the more frequent symptoms (Table 2). Hospitalization was required for 49.4% of patients and 22.9% received mechanical ventilation (Table 1). Baseline characteristics, the primary causes of ESRD, coexisting disorders, and dialysis modality were similar between the first and second waves (Table 1).

**Table 1.** Demographic characteristic and outcomes of patients with COVID-19 stratified by the first and second waves.

Characteristics	Total (*n* = 83)	First wave (*n* = 44)	Second wave (*n* = 39)	*p* Value
Age (years), mean (SD)	60 ± 15	57.5 ± 17	63.5 ± 12	0.08
Sex, male [*n* (%)]	49 (59)	25 (56)	23 (59)	1
Race (White/non-White)	65/18	34/10	31/8	0.9
Smoker [*n* (%)]	4 (4.8)	2 (4.5)	2 (5.1)	1.0
**Positive contact [*n* (%)]**				
Public transportation	11 (13)	8 (18)	7 (18)	1.0
Family members at home	30 (36.1)	18 (40.9)	21 (54)	0.27
During hospitalization	4 (4.8)	4 (9)	4 (10.2)	1.0
Does not know	12 (14.5)	5 (11.3)	7 (18)	0.53
**Primary causes of ESRD [*n* (%)]**				
Diabetes mellitus	33 (39.7)	13 (29.5)	20 (51.3)	0.07
Arterial hypertension	25 (31.1)	17 (38.6)	8 (20.5)	0.11
Others	25 (30.1)	14 (31.8)	11(28.2)	0.7
**Coexisting disorder [*n* (%)]**				
Cardiovascular disease	37 (44.6)	19 (43.1)	18 (46)	0.95
Arterial hypertension	78 (93.9)	39 (88.6)	38 (97.4)	0.26
Diabetes mellitus	44 (53)	21 (47.7)	23 (59)	0.42
Lung disease	21 (25.3)	13 (29.5)	8 (20.5)	0.49
Cancer	8 (6.6)	5 (11.3)	3 (7.7)	0.84
**Dialysis modality [*n* (%)]**				
Hemodialysis	70 (84.3)	40 (90.9)	30 (77)	0.14
Peritoneal dialysis	13 (15.6)	4 (9)	9 (23)	0.14
Dialysis (months), mean (SD)	45.6 ± 42	44 ± 46	39 ± 31.7	0.87
**Treatments [*n* (%)]**				
Oxygen therapy	38 (45.7)	17 (38.6)	21 (53.8)	0.16
Mechanical ventilation	19 (22.9)	**6 (13.6)**	**13 (33.3)**	**0.03**
Hospitalization [*n* (%)]	41 (49.4)	20 (45.4)	21 (53.8)	0.51
Hospitalization period (days), mean (SD)	6.2 ± 8.5	5.8 ± 8.7	6.6 ± 8.4	0.42
Intensive care unit [*n* (%)]	23 (27.7)	10 (22.7)	13 (33.3)	0.33
Incidence rate/10,000^a^	1762.2	997.7	1083.3	0.72
Mortality rate/10,000^a^	403.4	158.7	333.3	0.16
Fatality rate (%)^a^	22.9	**15.9**	**30.7**	**0.03**
Nonsevere disease [*n* (%)]	56 (67.5)	34 (77.2)	22 (56.4)	0.06
Severe disease [*n* (%)]	27 (32.5)	10 (22.7)	17 (43.6)	**0.04**
Death [*n* (%)]	19 (22.9)	**7 (15.9)**	**12 (30.7)**	**0.03**

^a^Calculations: The incidence, mortality, and case fatality rates were calculated as follows: Incidence = number of COVID-19 cases/ number of exposed people (dialysis patients) per 10,000. Mortality = number of deaths due to COVID-19/number of exposed people (dialysis patients) per 10,000. Fatality = (number of confirmed deaths due to COVID-19/number of confirmed COVID-19 cases) *100.

Bold values mean *p* < 0.05 and considered indicative of statistical significance.

**Table 2.** Symptoms, exams, and vaccination characteristics of patients with COVID-19 stratified by the first and second waves.

	Total (*n* = 83)	First wave (*n* = 44)	Second wave (*n* = 39)	*p* Value
**Symptoms [*n* (%)]**				
Fever	54 (65)	31 (70.4)	23 (52.2)	0.38
Cough	52 (62.6)	24 (54.5)	27 (6,2)	0.25
Dyspnea	44 (53)	23 (52.2)	21 (47.7)	0.89
Odynophagia	14 (16.8)	8 (18.1)	6 (15.4)	0.96
Diarrhea	19 (22.8)	**15 (34.0)**	**4 (10.2)**	**0.02**
Headache	11 (13.2)	9 (20.4)	2 (5.1)	0.08
Loss of taste or smell	23 (27.8)	**22 (50)**	**1 (2.56)**	**0.0001**
Asthenia	30 (36.1)	14 (31.8)	16 (41)	0.52
**Chest computer tomography scan ground glass lesions [*n* (%)]**	**(*n* = 60)**	**(*n* = 35)**	**(*n* = 25)**	
<25%	14 (23.3)	11 (31.4)	14 (56)	0.1
25–50%	30 (50)	17 (48.6)	8 (32)	0.30
>50%	9 (15)	7 (20)	3 (12)	0.63
**Laboratory findings, median (IQR)**				
Hemoglobin (g/dL)	10.6 ± 2	10.4 ± 2	10.9 ± 2	0.31
Platelet (per mm^3^)	182 ± 118.6	173.6 ± 89	193 ± 149	0.94
Leukocytes (per mm^3^)	5310 ± 2440	5380 ± 2461	5223 ± 2446	0.80
Lymphocytes (per mm^3^)	952 ± 563	**1091 ± 613**	**771 ± 436**	**0.01**
Neutrophils (per mm^3^)	3748 ± 2203	3927 ± 2186	3611 ± 2231	0.35
Neutrophil lymphocyte ratio	6.24 ± 10	**4.6 ± 5.5**	**8.38 ± 13.8**	**0.006**
C-reactive protein (mg/dL)	8.76 ± 10.5	8.9 ± 10.3	8.5 ± 10.6	0.94
Glutamic oxalacetic transaminase (U/L)	29.5 ± 30.4	28.8 ± 35.7	30.2 ± 21	0.77
Glutamic-pyruvic transaminase (U/L)	22 ± 29	21.3 ± 36	20.7 ± 14.3	0.92
Total serum bilirubin (mg/dL)	0.31 ± 0.22	0.32 ± 0.2	0.30 ± 0.23	0.78
Gamma GT (UI/L)	715.1 ± 248.6	127.3 ± 316.4	93 ± 87.5	0.54
Alkaline phosphatases (UI/L)	130 ± 111	145.6 ± 135	109.8 ± 51.6	0.33
D-dimer (Ug/mL)	2.12 ± 2.6	2.4 ± 3.1	1.64 ± 1.3	0.2
Lactic dehydrogenase (U/L)	303 ± 114	239 ± 109	312.7 ± 120	0.52

Bold values mean *p* < 0.05 and considered indicative of statistical significance.

In the first wave 44/441 patients while 39/360 patients in the second wave period, were diagnosed with COVID-19 disease. None of the infected patients has received the two doses of vaccines during the study period, and 19 (24%) of the patients were partially vaccinated during the second wave with ChAdOx1 nCoV-19 vaccine (AZD1222) or CoronaVac (Sinovac Biotech) vaccines.

As shown in Table 2, on admission, symptoms such as diarrhea and the loss of taste or smell were more frequent in the first wave group. Lymphopenia and higher neutrophil-to-lymphocyte ratios (N:L), the number of severe cases, MV need, and deaths occurred more frequently during the second wave.

In our study, the second wave compared with the first wave presented numerical but not statistically significant higher incidence (1083.3 vs. 997.7 per 10,000) and mortality rates (333.3 vs. 158.7 per 10,000); of note, we encountered significant differences in relation to the severity of the disease (43.6% vs. 22.7%; *p* = 0.04), case fatality rate (30.7% vs. 15.9%; *p* = 0.03), and the incidence rate per 100 patient-months (50.6 vs. 23.5; *p* < 0.001).

In our dialysis unit, the first case of COVID-19 among dialysis patients was detected on 26 May 2020, and the number of cases peaked in July 2020 (first wave) and the second wave peaked in March 2021, with a slight delay in relation to the spread of the disease in Brazil [3]. During the second wave infection period, the *Gamma* variant became the more prevalent variant in our region and Brazil [5–7].

Another study show that a higher rate of hospitalization and MV need was identified in the second wave in relation to the first wave, in addition, the variant *Gamma* in Manaus (Amazonas state) was associated with the increase in mortality of individuals between 20 and 59 years old [4,5].

Lymphopenia and higher N:L ratio were significantly higher in the second wave group, in agreement to a study that found that the N:L ratio was the major marker associated with severe forms and predicts short-term COVID-19 outcomes in hemodialysis patients [8], and lymphopenia was associated with severe pneumonia in another study [9].

Among limitations of our study, the single center design, the relatively low number of patients, and the absence of information regarding each patient’s virus sequence. In addition, not all patients in the dialysis station were tested; therefore, the actual number of infections in the unit cannot be given. Also, patients with natural immunity after SARS-CoV-2 infection and immunity after partial vaccination may influence the results of the study.

In conclusion, in our study, maintenance dialysis patients presented more severe disease with higher case fatality rate for SARS-CoV-2 infection during the second wave of the infection. We suggest that the highest severity of COVID-19 infection may be associated with the change of SARS-CoV-2 variants during the second wave period.

To our knowledge, this is the first study reporting the highest severity of COVID-19 infection associated with the change of SARS-CoV-2 variants in dialysis patients.
